# Comparative Analysis of Gut Microbiota in Patients with Irritable Bowel Syndrome and Healthy Controls

**DOI:** 10.3390/jcm14041198

**Published:** 2025-02-12

**Authors:** Pedro Sánchez-Pellicer, José María Álamo-Marzo, María Martínez-Villaescusa, Eva Núñez-Delegido, José Francisco Such-Ronda, Francisco Huertas-López, Emilio Manuel Serrano-López, David Martínez-Moreno, Vicente Navarro-López

**Affiliations:** 1 MiBioPath Research Group, Faculty of Medicine, Universidad Católica San Antonio de Murcia, 30107 Murcia, Spain; pedro.sanchez@bioithas.com (P.S.-P.); alamomarzojm@gmail.com (J.M.Á.-M.); dramariamvillaescusa@gmail.com (M.M.-V.); eva.nunez@bioithas.com (E.N.-D.); 2 Department of Clinical Laboratory, Hospital General Universitario Dr. Balmis, 03010 Alicante, Spain; 3 Department of Gastroenterology, Hospital Vithas Medimar, 03013 Alicante, Spain; josesuch.md@gmail.com; 4 Marbyt—Smart Solutions for Biotechnology S. L., 30100 Murcia, Spain; francisco.huertas@marbyt.com (F.H.-L.); emiliomanuel.serrano@marbyt.com (E.M.S.-L.); david.martinez@marbyt.com (D.M.-M.)

**Keywords:** irritable bowel syndrome, microbiota, gastrointestinal microbiome, functional colonic diseases, high-throughput nucleotide sequencing

## Abstract

**Background/Objectives**: Growing evidence highlights the pivotal role of gut dysbiosis in the pathophysiology of irritable bowel syndrome (IBS). Despite this, the identification of an “IBS microbiota signature” remains elusive, primarily due to the influence of genetic, dietary, and environmental factors. To address these confounding variables, it is critical to perform comparative analyses using a control group derived from the same community as the IBS patients. This study aimed to evaluate and contrast the gut microbiota composition of IBS patients with healthy controls. **Methods**: We compared the gut microbiota from stool samples of 25 IBS patients diagnosed according to the Rome IV criteria, and 110 healthy subjects without acute or chronic diseases and not on continuous medication. The high-throughput sequencing of the V3–V4 regions of the *16S rRNA* gene was conducted for microbiota analysis. **Results**: The IBS gut microbiota was richer but exhibited lower alpha diversity compared to the control group, suggesting simplification and imbalance. A beta diversity analysis revealed overall compositional differences between the two groups. A heat tree analysis highlighted key IBS-associated changes, including a decrease in Firmicutes, mainly due to Clostridia, and an increase in Bacteroidota, driven by an expansion of Bacteroidales families. Differential expression analyses identified important genera within these taxa like *Bacteroides*, *Faecalibacterium*, and *Blautia*, which could serve as microbiota-based biomarkers for IBS. **Conclusions**: Our results reveal both statistically and clinically significant differences in gut microbiota composition and diversity between IBS patients and healthy controls from the same community. These findings provide a deeper understanding of how alterations in the gut microbiota may contribute to IBS symptoms, offering new insights into the diagnosis and potential treatments.

## 1. Introduction

Irritable bowel syndrome (IBS) is classified as a functional gastrointestinal disorder, primarily characterized by recurrent abdominal pain accompanied by changes in bowel habits, either constipation, diarrhea, or a combination of both [1]. The Rome IV diagnostic criteria, effective since 2016, state that abdominal pain must occur at least once per week over the past three months, alongside specific criteria related to stool frequency and form, which must also be met during the past three months (with symptoms having started at least six months earlier) [2]. These criteria also distinguish the different subtypes of IBS according to the predominant stool pattern: constipation-predominant (IBS-C); diarrhea-predominant (IBS-D); with mixed bowel habits (IBS-M); unclassified (IBS-U); post-infectious IBS (specifically defined in 2019) [3]. This international consensus has enabled a positive diagnosis based on symptoms, moving away from the previously predominant approach of diagnosis by exclusion.

The pathophysiology of IBS is not fully elucidated. However, IBS is considered a biopsychosocial disorder characterized by abnormal brain–gut communication, which is exacerbated by various external and internal triggers [4,5,6]. Key biological factors involved include gut motility abnormalities [7], visceral hypersensitivity (either due to stress-induced hypervigilance or true hyperalgesia caused by sustained bowel stimulation) [8,9], and alterations in brain regions responsible for processing visceral stimuli, which reduce antinociceptive mechanisms for visceral pain, thereby amplifying its perception [5]. Additionally, a distorted hypothalamic–pituitary–adrenal axis response occurs, involving the primary mediator of the stress response corticotropin-releasing factor [10], as well as the activation of the immune system at the gut mucosal level, which leads to an increase in mast cells and intraepithelial lymphocytes, resulting in low-grade inflammation [11], along with defects in serotonergic signaling [12]. It is likely that a combination of these factors contributes to the manifestation of the variable symptoms. This variability suggests that various mechanisms could predominate in different subjects. Moreover, the current IBS conceptual model considers the role of psychosocial factors in increasing susceptibility to developing and exacerbating symptoms through brain–gut interactions.

In recent years, the gut microbiota has been identified as an integral player in gut–brain communication through multiple mechanisms. Signals from gut microbiota can modulate the gut–brain axis at neuronal, endocrine, and immune levels [13]. Furthermore, growing evidence supports that the gut microbiota is involved in the pathophysiology of IBS. Dysbiosis, along with compromised intestinal barrier function and low-grade inflammation, influencing even the activity of the enteric nervous system, are also key factors in the development of the IBS-associated symptomatology described above [11,14]. Systematic reviews of descriptive studies using next-generation sequencing (NGS) of bacterial *16S rRNA* gene have highlighted differences in the gut microbiota of IBS patients compared to healthy controls [15,16]. Nevertheless, identifying a distinct “IBS microbiota pattern” remains challenging.

The composition of the human gut microbiota is highly variable and is influenced by multiple factors such as ethnicity, geographic location, diet, infections, medications, and other host genetic-related, environmental, and lifestyle factors. In addition, different bacteria often share metabolic capabilities, leading to a multifunctional redundancy that introduces significant variability at a compositional level. Compositional variability complicates the definition of a healthy gut microbiota, a concept that remains currently under discussion [17]. Therefore, when analyzing the gut microbiota characteristics of IBS patients, it is crucial to compare them with healthy controls from the same community. This approach minimizes genetic, geographic, dietary, and environmental variability, increasing the chance that observed differences are attributable to IBS rather than other factors. This facilitates the identification of specific IBS-associated changes.

The characterization of the gut microbiota associated with IBS using best practices will clarify a key pathophysiological factor, potentially leading to the development of targeted therapies. Due to the significant impact of this disease [18], this represents a crucial research area. For all these reasons, the objective of this study was to compare the gut microbiota of IBS patients with that of healthy controls from the same community.

## 2. Materials and Methods

### 2.1. Study Design and Enrolled Population

The study employed a retrospective observational design, analyzing previously collected data.

The gut microbiota data were obtained from the stool samples of 25 patients with IBS and 110 healthy controls, all of whom participated in two previous clinical trials (registered at https://clinicaltrials.gov (accessed on 5 January 2025) with codes NCT05352724 and NCT05565612, respectively) conducted between 2022 and 2023 by our research team. The participants were recruited from the MiBioPath research team’s facilities at the Universidad Católica San Antonio de Murcia (Murcia, Spain).

The inclusion criteria for IBS patients were as follows: (I) age between 18 and 65 years, (II) diagnosis according to the Rome IV criteria. In this study, individuals were excluded if they (I) received antibiotic treatment in the 2 weeks prior to the beginning of the trial, (II) consumed probiotics in the 2 months prior to the beginning of the trial, (III) had inflammatory bowel diseases concurrent with IBS, and/or (IV) were pregnant or breastfeeding. Moreover, IBS patients were characterized based on their subtype (IBS-D, IBS-C, or IBS-M) [2], and severity as assessed by the “IBS Severity Scoring System” (IBS-SSS) [19].

In the other hand, controls were individuals between 18 and 65 years who practiced high-intensity aerobic exercise 3 to 5 times per week. Exclusion criteria for these healthy controls included the following: (I) chronic disease, (II) acute disease within 3 months prior to the study requiring corticosteroid treatment, (III) continuous pharmacological treatment, (IV) habitual drug or alcohol use, (V) antibiotic treatment in the 15 days prior to the study, (VI) probiotic intake in the 15 days prior to the study, and (VII) pregnancy or breastfeeding.

Both clinical trials, from which the study samples were obtained, were approved by the Institutional Review Board of Universidad Católica San Antonio de Murcia (Murcia, Spain) (approval code: CE042202) and Hospital Universitario del Vinalopó (Elche, Spain) (approval code: CEImHUV 2022.034), respectively, and were conducted in compliance with the Declaration of Helsinki. Written informed consent was obtained from all study participants.

### 2.2. Gut Microbiota Profiling

All stool samples were collected in a nucleic acid stabilizing solution, such as RNAlater (Thermo Fisher Scientific, Waltham, MA, USA) and subsequently stored at −20 °C until further processing. To perform the phylogenetic characterization of the gut microbiota, bacterial DNA was extracted from stool samples using the QIAamp PowerFecal Pro DNA Kit (Qiagen, Hilden, Germany). DNA concentration was quantified using the Qubit 4 Fluorometer (Thermo Fisher Scientific, Waltham, MA, USA), while its purity was evaluated with a NanoDrop spectrophotometer (Thermo Fisher Scientific, Waltham, MA, USA).

Thereafter, PCR amplification and high-throughput sequencing of the V3 and V4 variable regions of the bacterial *16S rRNA* gene were performed according to the “16S Metagenomic Sequencing Library Preparation protocol” for the Illumina MiSeq System (Illumina, San Diego, CA, USA). Basically, this standardized and widely recognized protocol for the study of the microbiome includes the following steps: (I) PCR amplification of the V3 and V4 variable regions of the *16S rRNA* gene using specific primers, (II) preparation for sequencing of amplified DNA by addition of adapters and normalization of amplicon libraries, and (III) sequencing of the prepared libraries to obtain the FASTQ file with the raw sequences.

### 2.3. Processing of the 16S rRNA Sequence Data

Gut microbiota data from all included participants were initially provided in FASTQ format as raw sequences. However, following the raw data processing outlined below, 7 samples from the control group were discarded.

As a starting point, the quality of the FASTQ files was evaluated using the FastQC software (version 0.12.0). Reads with a Phred score below 20 were considered unsuitable for further analysis. The overall quality of the sequences was deemed satisfactory, and no preliminary trimming was required. Raw data were then imported into the QIIME2 software (version 2024.10) [20] to process the workflow leading to the final taxonomical assignment of the sequences. Primarily, the integrated DADA2 algorithm [21] was used for denoising. The sequencing quality significantly declined after 230 bp, so the last 70 bp were truncated to improve the overall quality of the reads. This was followed by the application of a clustering algorithm to group similar sequences into operational taxonomic units (OTUs) with a 97% similarity threshold. Subsequently, taxonomic classification of the OTUs was then assigned based on the SILVA database [22], utilizing a Naive Bayes classifier from the scikit-learn tool. Finally, a table was generated that included the number of assigned sequences (and their corresponding relative abundances) across the different taxonomic levels for each sample.

At this stage in the process, gut microbiota data from both study groups were available for assessment and comparison using the MicrobiomeAnalyst web platform (version 2.0) [23]. After importing the gut microbiota data, an initial filter was applied to exclude OTUs with fewer than 4 read counts or those present in fewer than 10% of the samples. Additionally, OTUs showing less than 5% variability were excluded. A normalization step was performed to account for differences in library sizes across samples. This normalization was achieved using the Trimmed Mean of M-values (TMM) data transformation method.

### 2.4. Reporting Results and Statistical Analysis

Baseline clinical and demographic characteristics of all included patients were summarized. Continuous variables were expressed as mean and standard deviation, and categorical variables as proportions. For microbiome analysis, several biostatistical tools were used, as described below.

#### 2.4.1. Alpha and Beta Diversity Analysis

Alpha diversity at a genus taxonomic level was assessed using Shannon, Simpson, and Chao1 indices. A *t*-test was applied to evaluate the presence of statistically significant differences between study groups with respect to these indices (*p* < 0.05). Rarefaction curves of samples from IBS patients and healthy controls were generated to assess richness and the completeness of sampling.

Beta diversity assessment was presented as a Principal Coordinates Analysis (PCoA) plot constructed using the Bray–Curtis distance as a dissimilarity metric. In addition, statistically significant differences in Bray–Curtis, Jaccard, and Jensen–Shannon distances between the study groups were examined through a PERMANOVA test (*p* < 0.05). A dendrogram was also constructed using the Bray–Curtis distance and applying the Ward algorithm to classify the samples from both study groups and to highlight potential compositional similarities.

#### 2.4.2. Description of Gut Microbiota of IBS Patients and Healthy Controls and Visual Exploratory Methods

Barplots illustrating the distributions at the phylum, family, and genus levels were performed to explore the composition of the gut microbiota in IBS patients compared to healthy controls. In addition, a comparison of relative abundances at the family level between groups was also visualized using a heat tree, highlighting only taxa with a *p* < 0.05 after applying the Wilcoxon test. Moreover, to investigate the core microbiome, genera with a relative abundance of 0.01% or higher, consistently present in at least 20% of the samples across all groups, were identified and illustrated. These exploratory analyses were performed using the Metacoder package [24].

#### 2.4.3. Differential Abundance Analysis of Microbial Taxa

The Linear Discriminant Analysis Effect Size (LEfSe) tool was utilized to detect taxa with differential compositions [25], integrating statistical significance testing (e.g., Kruskal–Wallis) with biological significance evaluations (e.g., Linear Discriminant Analysis, LDA) to identify the taxa that most strongly indicated the differences between the study groups. The reference parameters used to identify a genus as statistically significant biomarker were an adjusted *p* < 0.05 and an LDA score ≥ 2. Additionally, the Random Forest machine learning algorithm was also applied to identify bacterial genera that distinguish between two datasets.

#### 2.4.4. Correlation Network Analysis

The Sparse Correlations for Compositional Data (SPARCC) algorithm was used to infer correlations between bacterial families in both IBS patients and healthy controls, using a workflow that incorporated the iNAP pipeline [26]. The representation and analysis of these ecological correlation networks were performed using the Cytoscape software (version 3.10.3) [27] with the Cytohubba tool [28] employed to identify the position and interactions of the different nodes. In this context, the topological analysis algorithms Degree and Maximal Clique Covers (MCC) were used to identify the most important nodes based on the number of interactions. Those nodes shared by both algorithms were considered hub nodes. Correlations deemed significant by the SPARCC algorithm must have a *p* < 0.1 and a correlation magnitude of ±0.3.

## 3. Results

### 3.1. Clinical and Demographic Data of Participants

Table 1, Table 2 and Table 3 present a detailed overview of the clinical and demographic characteristics of the study participants, which include 25 patients diagnosed with irritable bowel syndrome (IBS) and 103 healthy controls. In contrast to the control group, which had a nearly balanced sex ratio with approximately equal numbers of males and females, the IBS group was predominantly composed of females. Patients with IBS were primarily diagnosed with the IBS-D subtype and exhibited a moderate to severe symptomatology.

### 3.2. Alpha Diversity of Gut Microbiota in IBS Patients

**Figure 1:**
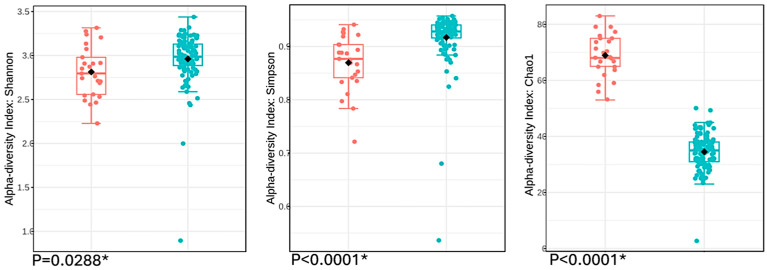
Boxplots of alpha diversity indices. Boxplots of alpha diversity indices revealed statistically significant differences between the mean values of patients with IBS (red) and healthy controls (blue), as assessed using the *t*-test. * Statistically significant difference.

**Figure 2:**
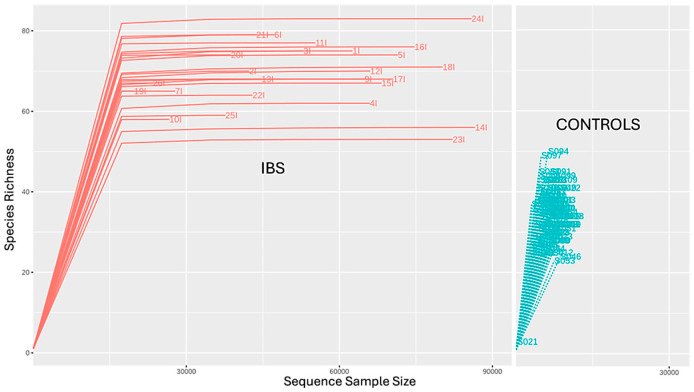
Rarefaction curves. Rarefaction curves of samples from IBS patients (red) and healthy controls (blue).

An analysis of the Chao1 estimator (Figure 1) revealed that the gut microbiota of IBS patients showed a higher richness compared to healthy controls, a finding that was also reflected in the rarefaction curve (Figure 2). However, a decrease in Shannon and Simpson indices (Figure 1) was observed in IBS patients compared to healthy controls. These insights indicated that while the gut microbiota of IBS patients was richer, it was less evenly distributed and less balanced than that of healthy controls.

### 3.3. Differences in Microbial Community Clustering Between IBS Patients and Healthy Controls

**Figure 3:**
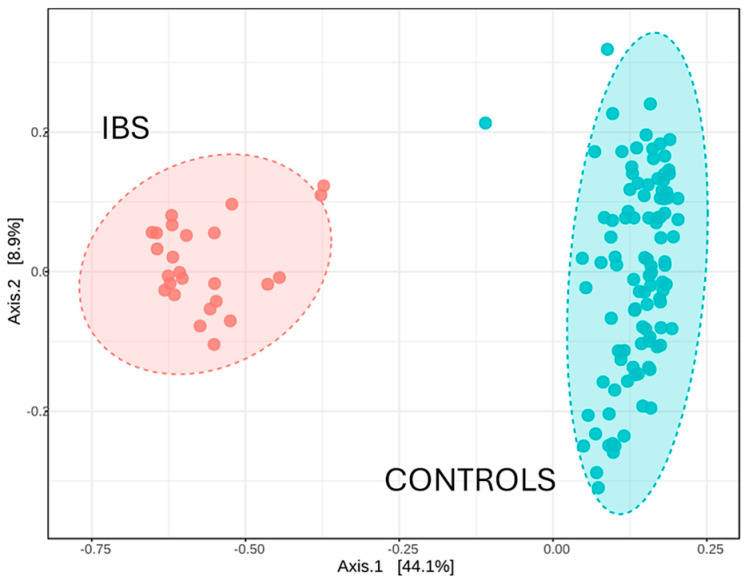
PCoA based on Bray–Curtis distance. PCoA based on Bray–Curtis distance from IBS patients (red) and healthy controls (blue). PCoA, Principal Coordinates Analysis.

**Figure 4:**
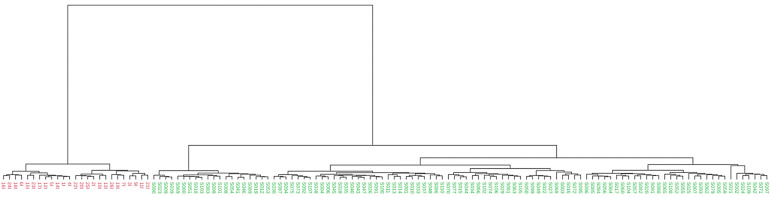
Dendrogram. Clustering dendrogram displaying distinct separation of IBS patients (red) and healthy control samples (green).

A significant dissimilarity in microbial community clustering between samples from IBS patients and healthy controls was observed through PCoA using the Bray–Curtis distance (Figure 3). This analysis revealed distinct clustering patterns, indicating substantial differences in gut microbiota composition between both study groups. Table 4 summarizes the statistically significant differences detected in Bray–Curtis, Jaccard, and Jensen–Shannon distances between the study groups, as determined by the PERMANOVA method. Furthermore, a dendrogram also constructed using the Bray–Curtis distance corroborated the classification of the samples according to the health status of the participants, distinguishing between healthy and IBS-affected individuals (Figure 4).

### 3.4. Compositional Differences at Phylum, Family, and Genus Levels Between Patients with IBS and Healthy Controls Evidenced by Visual Exploratory Methods

**Figure 5:**
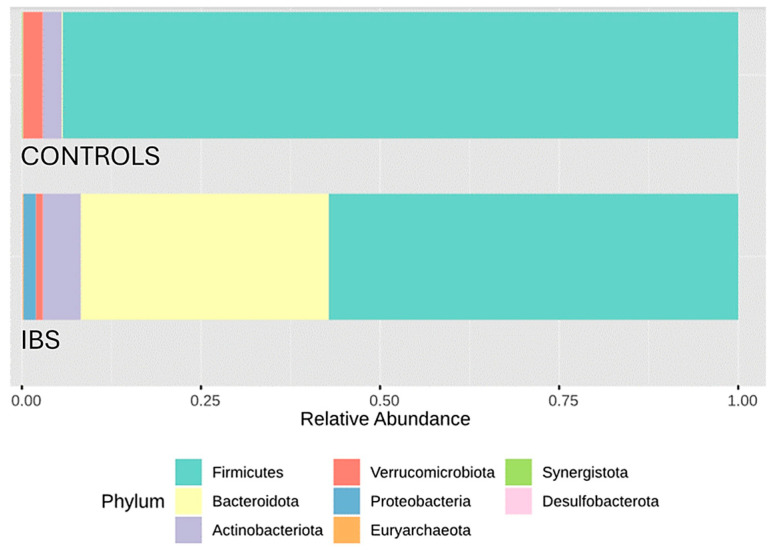
Barplot of relative abundances of major phyla in patients with IBS and healthy controls. Distribution of the relative abundances of the main phyla in the study groups. The colored bars represent the mean proportion of each phylum relative to the total gut microbiota composition in each group. Each color corresponds to a specific phylum, as indicated in the legend.

**Figure 6:**
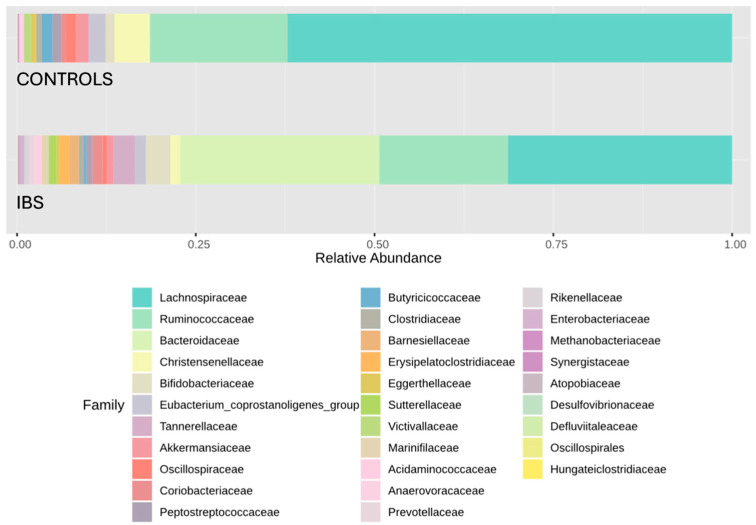
Barplot of relative abundances of main families in patients with IBS and healthy controls. Distribution of the relative abundances of the main families in the study groups. The colored bars represent the mean proportion of each family relative to the total gut microbiota composition in each group. Each color corresponds to a specific family, as indicated in the legend.

**Figure 7:**
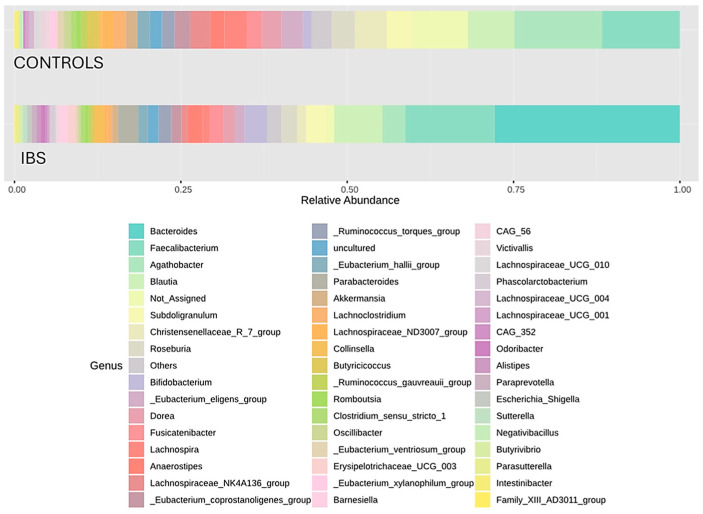
Barplot of relative abundances of main genera in patients with IBS and healthy controls. Distribution of the relative abundances of the main families in the study groups. The colored bars represent the mean proportion of each family relative to the total gut microbiota composition in each group. Each color corresponds to a specific family, as indicated in the legend.

A visual exploration of relative abundance distributions at the phylum, family, and genus levels reveals marked contrasts in microbial composition between IBS patients and healthy controls. At the phylum level, an inversion of the Firmicutes/Bacteroidota ratio in IBS patients compared to controls was particularly noteworthy, with a decrease in Firmicutes and an increase in Bacteroidota (Figure 5). The reduction in Firmicutes in IBS patients was primarily due to a depletion of Lachnospiraceae, with smaller contributions from decreases in Ruminococcaceae and Christensenellaceae families. The increase in Bacteroidota was mainly due to an expansion of the Bacteroidaceae family (Figure 6). At the genus level, this finding led to a strong presence of *Bacteroides* and marked reductions in *Agathobacter*, *Subdoligranulum*, and *Christensenellaceae R7 group* (Figure 7).

**Figure 8:**
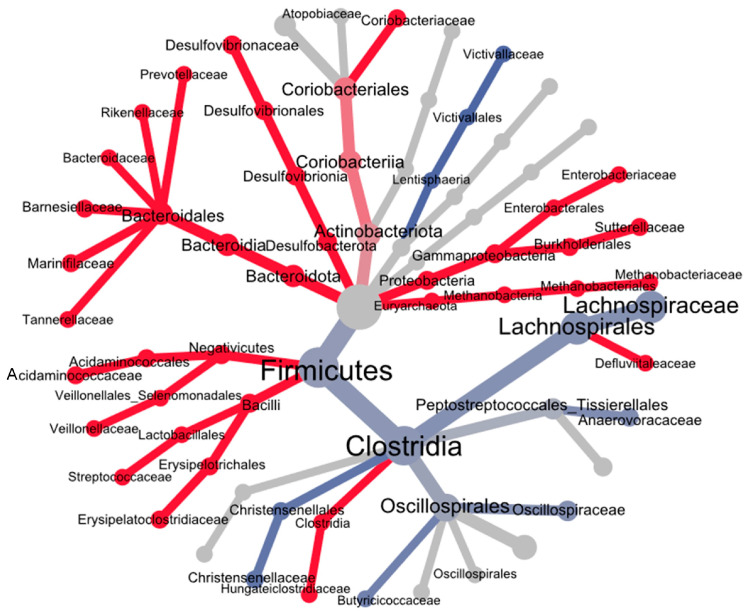
Heat tree of taxonomic differences. Hierarchical tree illustrating taxonomic differences between study groups. Nodes represent the taxonomic levels, and branches connect the nodes, indicating their hierarchical relationships. Colors depict the direction and magnitude of differences between groups: red (warm) denotes taxa more abundant in IBS patients, while blue (cool) indicates taxa more abundant in healthy controls. The intensity of the color reflects the magnitude of the difference, with darker shades representing greater differences. The color gradient, node size, branch thickness, and labels are determined based on the log2 ratio of mean abundance.

An examination of the heat tree expanded the insights provided by the previous barplots, revealing several significant differences in the gut microbiota composition between IBS patients and healthy controls (Figure 8). In this regard, a notable decrease in the relative abundance of Firmicutes primarily affected the Clostridia class and many of its constituent families. However, increases were observed in Bacilli and Negativicutes classes. Furthermore, Bacteroidota phylum exhibited an increase, primarily at the expense of families within the order Bacteroidales. Less abundant phyla such as Desulfobacterota, Proteobacteria, and Euryarchaeota also displayed increases. The heat tree analysis also highlights a pronounced decrease in the Victivallaceae family (Lentisphaerota phylum).

**Figure 9:**
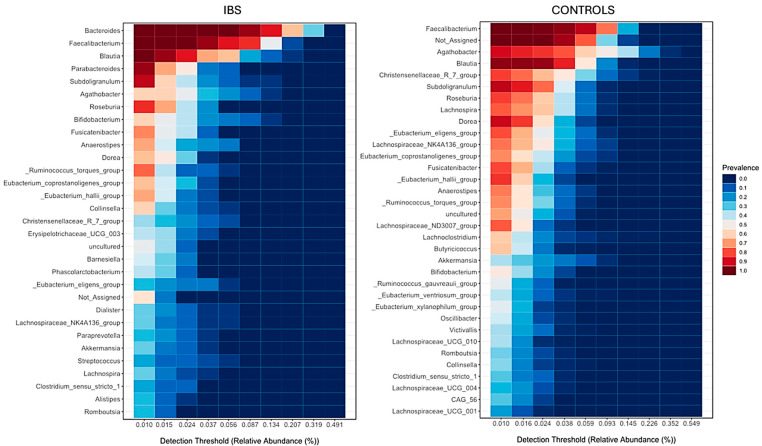
Core microbiome analysis. Higher levels of prevalence (red) indicate greater presence of these taxa in the samples, while low levels (blue) indicate little presence.

Graphs obtained from the core microbiome analysis (Figure 9) show that *Bacteroides* was the central genus in IBS patients, a pattern not observed in healthy controls. In healthy controls, the core microbiome was predominantly composed of genera from the phylum Firmicutes, including *Faecalibacterium* (which was the second most important genus in IBS patients), *Agathobacter*, *Blautia*, *Christensenellaceae R7 group*, *Subdoligranulum*, *Roseburia*, *Lachnospira*, and *Dorea*. Conversely, *Agathobacter*, *Christensenellaceae R7 group*, and *Lachnospira* lost relevance as core genera in IBS patients, while *Parabacteroides* emerged as a key genus. Despite these changes, genera belonging to Firmicutes still constituted a significant portion of the core microbiome in IBS patients.

### 3.5. Genera with Differential Expression Identified in IBS Patients as Potential Biomarkers

**Figure 10:**
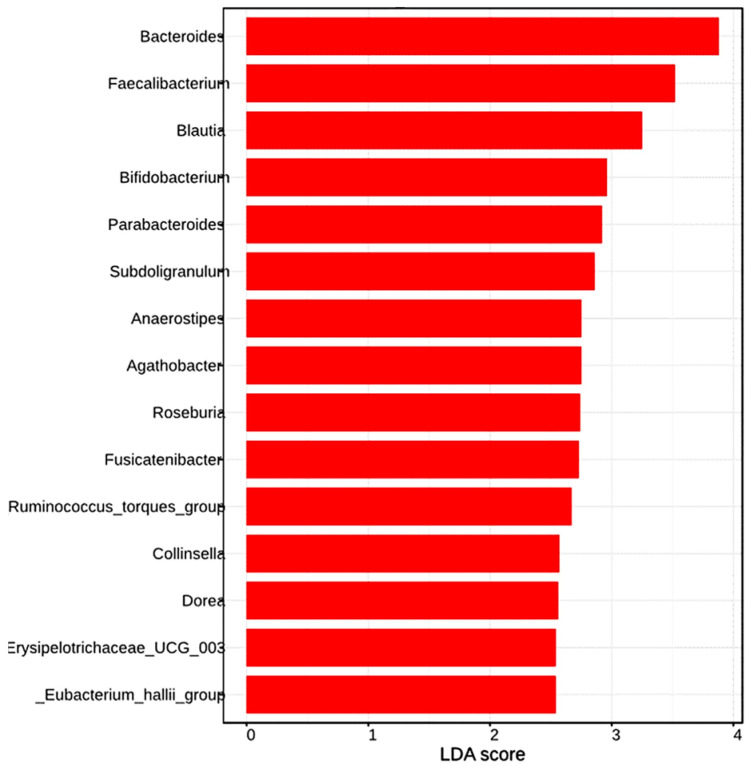
LEfSe. Genera identified as biomarkers by LEfSe in a comparison between IBS patients and healthy controls with LDA > 2. LEfSe, Linear Discriminant Analysis Effect Size; LDA, Linear Discriminant Analysis.

Figure 10 summarizes the genera identified by LEfSe as differentially expressed in IBS patients compared to healthy controls, with an LDA score > 2. Moreover, taxa with an LDA score > 3, which most significantly explain the differences in the gut microbiota between IBS patients and healthy controls, included *Bacteroides*, *Faecalibacterium*, and *Blautia*. Additional genera with LDA scores ranging from 2 to 3 were *Bifidobacterium*, *Parabacteroides*, *Subdoligranulum*, *Anaerostipes*, *Agathobacter*, and others.

**Figure 11:**
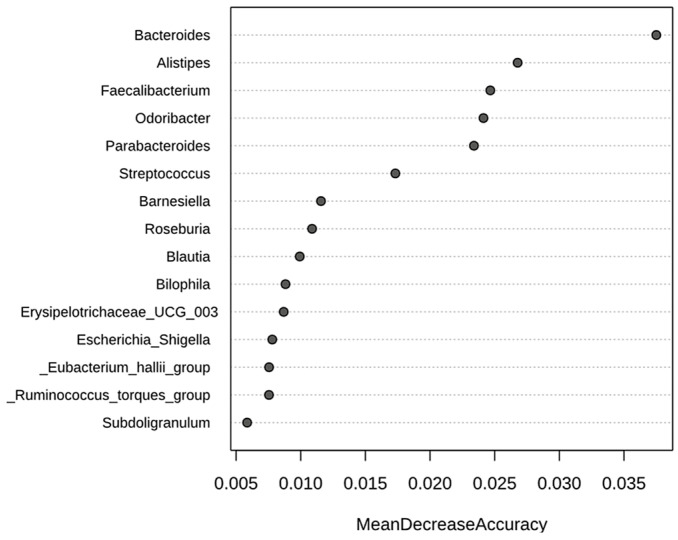
Random Forest. Genera identified as biomarkers by Random Forest in a comparison between IBS patients and healthy controls. “Mean Decrease in Accuracy” is a metric used in Random Forest models to evaluate the importance of different taxa in model predictions. The higher the positive value, the more crucial the feature is for the model.

In addition, Figure 11 highlights the genera that are especially characteristic of IBS patients, identified using the Random Forest algorithm. According to this model, the genera that most significantly contributed to the classification of samples as belonging to IBS patients, in descending order of importance, were *Bacteroides*, *Alistipes*, *Faecalibacterium*, *Odoribacter*, *Parabacteroides*, and *Streptococcus*. In this way, *Bacteroides*, *Faecalibacterium*, *Blautia*, *Parabacteroides*, *Subdoligranolum*, *Ruminococcus torques group*, *Erysipelotricaceae UGC 003*, and *Eubacterium halii group* were the genera identified by both LEfSe and Random Forest as characteristic and differentially expressed by IBS patients compared to healthy controls.

### 3.6. Correlation Network Analysis Identified Characteristic Interactions Between Families in Patients with IBS

**Figure 12:**
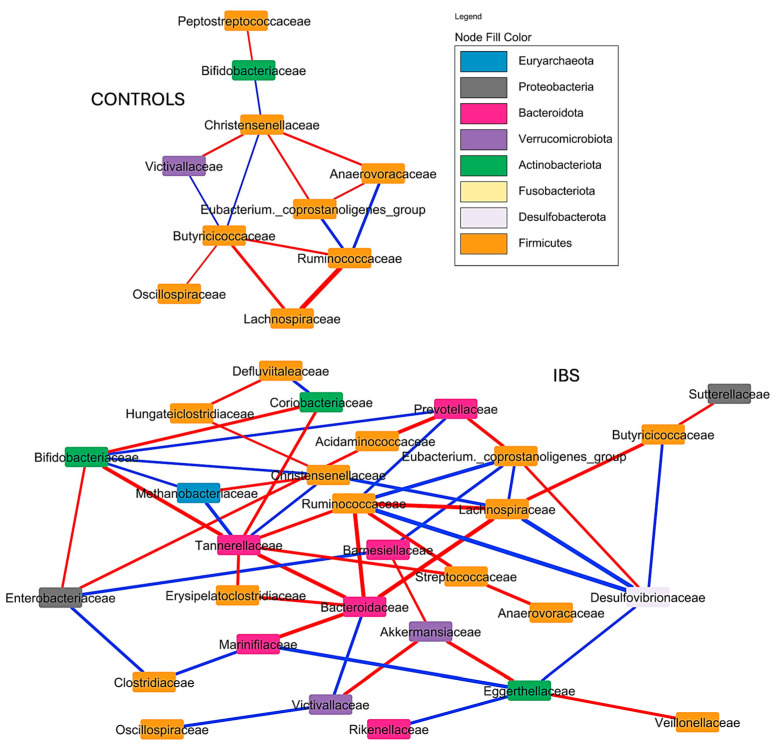
Correlation network analysis. Correlation network analysis at family level determined by the SPARCC algorithm in patients with IBS and healthy controls. Blue connections denote positive correlations, while red connections signify negative correlations. The width of the connection lines represents the strength of the correlation: wider lines indicate a stronger correlation between nodes, whether positive or negative. Correlations considered statistically significant were those with a *p* < 0.1 and a correlation magnitude of ±0.3. The nodes were colored according to their respective phylum, as indicated by the colors in the figure legend. SPARCC, Sparse Correlations for Compositional Data.

The correlation network analysis at the family level, as determined by SPARCC algorithm, revealed that families belonging to the phylum Bacteroidota play a central role in the gut microbiota of IBS patients. These families often served as nodes with numerous correlations, both positive and negative. Notably, they showed a tendency to exhibit predominantly positive correlations with other families of the same phylum, while displaying both positive and negative correlations with families from other phyla. Additionally, the Christensenellaceae family was identified as a key taxon in these patients, serving as the node with the most statistically significant correlations. Some notable negative correlations included those between Lachnospiraceae and Ruminococcaceae families with Bacteroidaceae. Conversely, significant positive correlations were observed between Bacteroidaceae and Victivallaceae, as well as between Lachnospiraceae and all three of Desulfovibrionaceae, *Eubacterium coprostaligenes group*, and Christensenellaceae. Figure 12 illustrates the full correlation networks for both healthy controls and IBS patients.

## 4. Discussion

This study clearly demonstrated that the gut microbiota of patients with IBS exhibited a range of distinct characteristics compared to the gut microbiota of healthy individuals from the same community. Firstly, the gut microbiota of IBS patients was richer but displays lower alpha diversity compared to that of the control group. This suggests a simplification in the distribution and balance of bacterial communities, where a smaller subset of bacteria became more dominant. Furthermore, a beta diversity analysis clearly revealed significant dissimilarities between stool samples from IBS patients and healthy controls, indicating notable differences in their gut microbiota composition. In this regard, the heat tree analysis highlighted specific changes. At the phylum level, IBS patients showed a reduction in Firmicutes and an increase in Bacteroidota. The reduction in Firmicutes primarily involved the Clostridia class, although there were noticeable increases in Negativicutes and Bacilli classes including families such as Acidaminocaccaceae, Veillonellaceae, Streptococacceae, and Erysipelotoclostridiaceae. The heat tree illustrated that the Lachnospiraceae family showed the most significant reduction in Clostridia members. Other families that displayed a decreased presence in IBS patients included Christensenellaceae, Anaerovoracaceae, Oscillospiraceae, and Butyricicoccaceae. On the other hand, the observed increase in the phylum Bacteroidota was primarily driven by the expansion of several families within the order Bacteroidales, including Bacteroidaceae, Tannerellaceae, Marinifilaceae, Barnesiellaceae, Rikenellaceae, and Prevotellaceae. The correlation network analysis revealed that the Bacteroidaceae family is a pivotal taxon, establishing numerous associations with other families. Notably, an inverse correlation was observed between Bacteroidaceae and short-chain fatty acid (SCFA)-producing families such as Lachnospiraceae and Ruminococcaceae. In addition, the heat tree analysis revealed significant increases in families from less prevalent phyla, such as Desulfovibrionaceae (Desulfobacteroidota), Enterobacteriaceae and Sutterellaceae (Proteobacteria), Methanobacteriaceae (Euryarchaeota), and Coriobacteriacea (Actinobacterota). Finally, a significant decrease in the Victivallaceae family within the phylum Lentisphaerota was also noteworthy. Consequently, differential expression analyses of the gut microbiota in IBS patients, utilizing tools such as LEfSe and Random Forest, identified several key genera, predominantly from the aforementioned families. These included *Bacteroides*, *Faecalibacterium*, *Blautia*, *Bifidobacterium*, *Parabacteroides*, *Subdoligranulum*, *Anaerostipes*, *Agathobacter*, *Roseburia*, *Fusocatenibacter*, *Ruminococcus torques group*, *Collinsella*, *Dorea*, *Erypelotrichaceae UCG-003*, *Eubacterium hallii group*, *Alistipes*, *Odoribacter*, *Streptococcus*, *Barnesiella*, *Bilophila*, and *Escherichia*. These genera could be integral to understanding the microbial shifts associated with IBS, as they likely play significant roles in the altered metabolic, immune, and inflammatory responses observed in these patients. Overall, the alterations observed in the gut microbiota of IBS patients suggest a state of dysbiosis, which may play a pivotal role in the pathophysiology of IBS by disrupting gut homeostasis, modulating immune responses, and intensifying gastrointestinal symptoms.

In the past 5 years, several systematic reviews of case–control studies comparing the gut microbiota of patients with IBS to that of healthy controls have been published [15,16,29,30,31]. These studies have demonstrated that the gut microbiota in IBS exhibits an altered state, with an increased dysbiosis index, particularly in cases of IBS-D [29]. However, the findings that characterize this dysbiosis state or the compositional deviations from healthy controls have been quite inconsistent. This inconsistency arises from several factors, such as the inclusion of patients with varying IBS subtypes and the heterogeneity in methods used to evaluate the gut microbiota [16,30]. Although most studies have utilized NGS methodologies targeting the *16S rRNA* gene, the amplified regions often vary, and different databases are used for taxonomic assignment, among other differences.

The IBS patients in our study exhibit a gut microbiota with lower alpha diversity compared to healthy controls (Figure 1). Similarly, the systematic review of Duan et al. revealed an overall trend of reduced alpha diversity associated with IBS, although discrepancies in some studies were noted [15]. Nevertheless, Pittayanon et al. also observed that while many studies reported reduced alpha diversity, other studies found no significant differences [16]. We consider that the rise in gut microbiota richness among IBS patients in our study is a notable finding that could have important therapeutic implications. However, this result has not been consistent across previous case–control studies, whether assessed as total bacterial species number or through the Chao1 richness estimator [15,16,31]. Recently, Su et al. found a significant difference but only regarding the IBS-D subtype in a large cohort of IBS patients, observing a reduction in total bacterial species compared to a matched group of healthy controls [32]. In this regard, the clinical guideline from the American College of Gastroenterology recommends rifaximin for the treatment of IBS-D symptoms, based on the hypothesis that these patients exhibit gut dysbiosis [33]. Our findings regarding alpha diversity and microbial richness support the notion of an imbalanced IBS-associated gut microbiota, which may potentially be restored with a non-absorbable antibiotic [34].

Concerning characteristic changes in specific members of the IBS gut microbiota, some authors have identified consistent trends. Contrary to our results (Figure 5), Duan et al. observed a decrease in Firmicutes (mainly the Clostridia class) and an increase in Bacteroidota (mainly the Bacteroidia class) [15], although another systematic review reported discrepant results [16]. A consistent finding in the literature is the increase in the phylum Proteobacteria, particularly within the Enterobacteriaceae family [15,16,31], which was also observed in our study (Figure 5). Moreover, several genus-level changes evidenced in our study aligned with previously reported findings. However, it is important to note that consistency across published systematic reviews remains limited. Notably, these changes underscore the loss of potentially beneficial genera, such as *Faecalibacterium* [16], *Lactobacillus* [31], and *Bifidobacterium* [16,31]. Nevertheless, despite belonging to the Clostridia class, *Faecalibacterium* retained a relatively high abundance in IBS patients (Figure 7). In fact, it was positioned as the second most influential genus based on the core microbiome analysis (Figure 9). Additionally, differential compositional analyses (Figure 10 and Figure 11) highlight *Faecalibacterium* as a characteristic biomarker in IBS patients. On the other hand, *Bacteroides* exhibited a marked and significant increase in patients with IBS, making it the most important genus (Figure 7). This observation is consistent with the findings of several systematic reviews [16], while others have reported inconclusive results [31] or even a decrease in its relative abundance [15]. It is interesting to note that the diagnostic clinical relevance of all these genera, which are potential microbiota-based biomarkers identified by the NGS of the *16S rRNA* gene, is really limited. In this regard, after identifying IBS-associated taxa by NGS methodology, the next steps should include the development of its quantitative PCR (qPCR) assay, followed by a new case–control study to validate its diagnostic accuracy. However, it is only in cases where qPCR assay’s diagnostic accuracy was found to be exceptionally elevated that a subsequent diagnostic accuracy study should be conducted in a real clinical setting to assess its practical usefulness [35].

Some previously mentioned systematic reviews have attempted to determine whether a specific gut microbiota signature is associated with the IBS subtype of the included subjects. The key question is whether there is a gut microbiota profile that differentiates the IBS-D and IBS-M subtypes. Whereas some authors have observed significant changes between the two subtypes in individual studies, these findings are inconsistent [15,16]. Teige et al. reported that only the gut microbiota of IBS-D patients showed significant differences compared to healthy controls. An increase in *Ruminococcus gnavus* was observed in these subjects in two out of the five studies included in their systematic review, which specifically used the GA-map^®^ Dysbiosis Test to characterize the gut microbiota [29]. In this regard, a large and comprehensive descriptive study has recently been published, examining gut microbiota signatures in 942 patients with IBS and comparing them with 942 non-IBS controls who were matched by age, sex, BMI, country, and diet [32]. Through PCoA and the PERMANOVA test of Bray–Curtis distances, patients with IBS-D, IBS-C, and IBS-U exhibited distinct compositional clustering patterns. Alpha diversity was decreased in patients with subtypes IBS-D and IBS-U but not with IBS-C, compared to controls. Additionally, an LEfSe analysis identified specific characteristics of the gut microbiota associated with each IBS phenotype when compared to the matched control group. Eleven genera exhibited contrasting trends across the different study groups. For example, genera such as *Subdoligranulum*, *Dorea*, *Eubacterium hallii group*, and *Haemophilus* were enriched in IBS-D but reduced in IBS-C. The authors also noted that many bacteria identified as enriched in IBS patients were potential pathogens. Specifically, *Ruminococcus gnavus* was associated with IBS-D, *Oscillibacter* with IBS-C, and *Ruminococcus torques* with IBS-U. In contrast, beneficial bacteria such as *Butyricoccus* in IBS-C, *Alistipes* in IBS-D, and *Turicibacter* in IBS-U were depleted. By employing an adequate sample size and controlling for confounding factors, this study identified bacteria and gut microbiota characteristics associated with each IBS subtype [32].

A notable limitation of our study is the lack of subanalyses based on IBS subtype. This limitation arose from the small sample size of the IBS group, with most patients belonging to the IBS-D subtype, which did not permit detailed stratification. Future descriptive studies on the gut microbiota of IBS patients should aim to encompass the broad clinical spectrum of the disease, including various subtypes and severities, to provide a more comprehensive understanding of the microbiota’s role in IBS. Given the heterogeneity of IBS, it is crucial to include diverse patient populations to capture the full range of microbiome characteristics that may correlate with different clinical presentations. In contrast, the current study is limited by a restrictive patient sample, which predominantly represents a specific subset of IBS patients, potentially overlooking the broader diversity of microbiota profiles that may be present across different IBS phenotypes. This limitation should be considered when interpreting the findings.

Although adopting a mechanistic approach based solely on the composition of the gut microbiota in IBS patients is challenging, some findings from our study were clinically significant and may be linked to specific pathophysiological processes. In this regard, there is evidence suggesting that pathogenic bacteria play a role in both the onset and exacerbation of symptoms in patients with IBS. These pathogens contribute to the activation and proliferation of immune cells involved in the pathophysiology of IBS, such as mast cells and T-cells, and promote the secretion of proinflammatory cytokines. These immune responses lead to increased intestinal permeability, alterations in intestinal motility, and the dysregulation of pain signaling [36]. Our study demonstrated that the microbiota of individuals with IBS was characteristic of a less balanced dysbiosis status, marked by the expansion of well-known pathogens such as Enterobacteriaceae. Furthermore, our IBS cases showed a significant depletion of SCFA-producing bacteria, with a pronounced reduction in members of the Lachnospiraceae (primarily) and Ruminococcaceae families, a mechanism likely contributing to the pathogenesis. Butyrate, the main SCFA, has a potent anti-inflammatory effect by inducing immune regulatory cells [37]. Consequently, the disrupted gut microbiota in IBS patients is likely to establish a proinflammatory microenvironment that exacerbates the dysfunction of the intestinal epithelium. On the other hand, the depletion of the Clostridia class and enrichment of the Bacteroidia class observed in the IBS gut microbiota should impact the composition and concentration of the bile acid pool. For instance, several *Bacteroides* species exhibit elevated bile salt hydrolase (BSH) activity, which deconjugates primary bile acids from taurine and glycine [38]. This alteration can affect key bile acid receptors, including the G-protein-coupled bile acid receptor 1 (TGR5) and the farnesoid X receptor (FXR), which are crucial for the proper functioning of the gut–brain axis. Recently, Zhan et al. have discussed how these changes in bile acid profiles influence the pathophysiology of the IBS-D subtype [38]. Finally, the increase in Methanobacteriaceae in our IBS cases was also noteworthy (Figure 8). Given that this family includes *Methanobrevibacter smithii*, its increase suggested an elevated CH_4_ production. Previous studies have shown that a higher presence of this bacterium in stool samples of IBS-C patients is associated with increased intestinal CH_4_ production, which contributes to alterations in intestinal motility [39]. Therefore, this finding could have significant implications for IBS-C patients.

The mechanistic hypotheses formulated based on the gut microbiota data could also be linked to therapeutic strategies aimed at modulating it. In this sense, probiotics may represent a promising approach for managing IBS, particularly if they prove effective in modulating key aspects of gut microbiota composition associated with IBS. A potential therapeutic benefit is their ability to inhibit the expansion of pathogenic microorganisms [40]. Moreover, probiotics could help restore a balanced gut microbiome by promoting the growth of beneficial bacteria that produce SCFA [41]. Additionally, probiotics may play a role in regulating the composition and concentration of the bile acid pool [42].

A key strength of this study was the inclusion of a large group of healthy controls from the same community population as the IBS cases, who were selected using strict criteria. This approach is crucial in microbiome comparative studies, as significant variations exist in microbial composition and functions encoded by the gut microbiota metagenome across different populations [43]. These differences, particularly evident in metabolic and immune pathways, reflect diverse environmental conditions, dietary habits, and lifestyles. Such variations can influence overall health and disease susceptibility. Therefore, to minimize variability related to geography, age, and environmental conditions, we carefully selected healthy controls that are demographically and environmentally like IBS cases. However, diet remained an uncontrolled factor, despite being a significant modulator of the gut microbiota [44]. Since most subjects in both groups were of normal weight, we assume that their diets are generally like the Mediterranean diet, characterized by a lower abundance of high-fat foods and ultra-processed products.

Women predominately constituted the IBS group, whereas the control group was more balanced in terms of sex distribution (Table 1). Limited studies have thoroughly examined the impact of sex on the composition of the gut microbiota, and the findings have generally been inconclusive and inconsistent [45]. For instance, research by Falony et al. identified sex as the 10th most significant variable influencing the composition of the gut microbiota across two cohorts [46]. This finding suggests that while sex may play a role, its impact is relatively minor compared to other factors that influence microbiota diversity and composition.

In summary, our results reveal both statistically and clinically significant differences in gut microbiota composition and diversity between patients with IBS and healthy controls from the same community. Specifically, the dysbiotic state of the IBS gut microbiota is reflected in an imbalance in terms of alpha diversity and distinct shifts in the abundance of numerous taxa. These findings provide a deeper understanding of how alterations in the gut microbiota may contribute to IBS symptoms. Identifying specific microbial patterns could offer new insights into the diagnosis and treatment of IBS. The results suggest that interventions aimed at restoring a healthy microbiota composition could be a promising therapeutic strategy for IBS patients. Additionally, personalized approaches to IBS management, based on individual microbial profiles, may open new avenues for treatment and diagnostic research.

## Figures and Tables

**Table 1:** Characteristics for included patients with IBS and healthy controls.

	IBS Patients (*n* = 25)	Healthy Controls (*n* = 103)
Sex (women)	21 (84.0%)	50 (48.5%)
Age (years)	43 (12)	37 (12)
Weight (kg)	64 (12)	72 (13)
Height (cm)	166 (6)	171(9)
BMI (kg/m^2^)	23.2 (3.6)	24.4 (2.9)

Continuous variables reported as mean (SD) and categorical variables presented as *n* (%).

**Table 2:** Clinical categorization of IBS patients according to their IBS subtype.

	IBS Subtype
IBS-D	14 (56%)
IBS-C	6 (24%)
IBS-M	5 (20%)

IBS-D, IBS diarrhea-predominant; IBS-C, IBS constipation-predominant; IBS-M, IBS with mixed bowel habits. Results presented as *n* (%).

**Table 3:** Clinical categorization of IBS patients according to their IBS-SSS stage.

	IBS-SSS
Severe	17 (68%)
Moderate	7 (28%)
Mild	1 (4%)

IBS-SSS, IBS Severity Scoring System. Results presented as *n* (%).

**Table 4:** PERMANOVA test for beta diversity distances.

	F-Value	R^2^	*p*-Value
Bray–Curtis	46.052	0.26766	0.001 *
Jaccard	28.423	0.18406	0.001 *
Jansen-Shannon	93.857	0.42690	0.001 *

* Statistical significance difference.

## Data Availability

The data from this study have not been deposited in a public repository due to confidentiality reasons related to commercial interests. However, raw sequence data can be directly requested from the corresponding author for scientific purposes.

## Abbreviations

The following abbreviations are used in this manuscript:

FXR	Farnesoid X Receptor
BSH	Bile Salt Hydrolase
LDA	Linear Discriminant Analysis
LEfSe	Linear Discriminant Analysis Effect Size
IBS	Irritable Bowel Syndrome
IBS-C	Irritable Bowel Syndrome Constipation-Predominant Subtype
IBS-D	Irritable Bowel Syndrome Diarrhea-Predominant Subtype
IBS-M	Irritable Bowel Syndrome with Mixed Bowel Habits
IBS-SSS	Irritable Bowel Syndrome Severity Scoring System
IBS-U	Irritable Bowel Syndrome Unclassified
MCC	Maximal Clique Covers
NGS	Next-Generation Sequencing
QOL	Quality of Life
PCOA	Principal Coordinates Analysis
SPARCC	Sparse Correlations for Compositional Data
TMM	Trimmed Mean of M-Values
